# Mood Disorder Due to a General Medical Condition with Manic Features

**DOI:** 10.1155/2009/871365

**Published:** 2009-07-01

**Authors:** J. P. Oliveira, S. L. Morais, D. Araújo, C. G. Carlotti, B. O. Colli, J. A. S. Crippa, J. E. Cecílio Hallak

**Affiliations:** Ribeirao Preto Medical School, University of Sao Paulo, das Clinicas - Terceiro Andar, Avenida Bandeirantes Campus Universitário, 3900 Ribeirao Preto, SP, Brazil

## Abstract

This case report describes a patient with manic and psychotic symptoms who had a history of neurocysticercosis and presented with an episode of hypertensive hydrocephalus in 2003. Despite her history, she was initially treated for primary psychiatric disease.

## 1. Introduction

Mrs. A, a female patient, 41 years old, was admitted in 2006 with agitation, attention deficit, temporal disorientation, exaggerated humor, logorrhea, grandiosity, mystic and somatic delusions, reduced need to sleep, and deficits of recent and immediate memories. She had been irritated, walking back and forth, talking alone, and praying uninterruptedly for three days. On the day of her admission, after abandoning her home, she was found singing and screaming religious hymns on the street.

## 2. Case Presentation

At the emergency service, she indicated that she had premonitions and was pregnant with eight twins. The physician noted “the patient is agitated and arrogant, thinking that I want to harm her and her “babies”. She believes that Jesus has already cured her and that she is here just to preach”.

Seven days after admission, she was referred to the inpatient clinic with a diagnosis of primary bipolar disorder. At this time, she was on risperidone 4 mg/d and valproate 1750 mg/d, without adequate clinical response. She felt special and believed that she had the capacity to speak with God, as evidenced in this passage: “Today is Tuesday, September 25, 1964. God spoke to me: Don’t fear, I am with you! I am crying of happiness. Today my husband will come to look for me. He is already coming … Jesus told me”.

**Figure 1:**
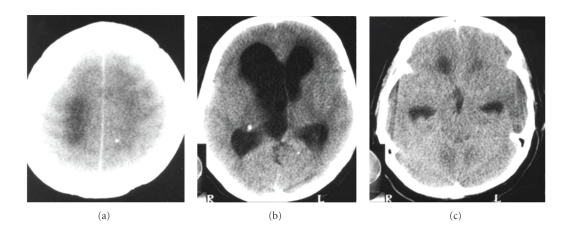
Noncontrast computed tomography (CT) scans (2003). (a) Parasagittal convexity. (b) Thalami. (c) Cerebral peduncles. There is ventricular enlargement with intracranial hypertension, and calcified nodules are scattered in the brain parenchima.

**Figure 2:**
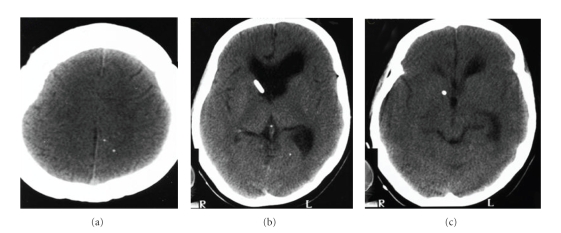
Noncontrast CT scans (2003). Ventriculo-peritoneal shunt. Calcified nodules are scattered in the parenchyma. There is improvement of intracranial hypertension, as compared to Figure 1.

In 2003, she had presented with mental confusion, agitation, psychosis, headache, vomiting, and weakness in her legs. She was diagnosed with hypertensive hydrocephalus (Figure 1) and submitted to a ventricle-peritoneal shunt (Figure 2). After three days, she remained agitated, aggressive, claiming to be possessed by the devil. She improved after being medicated with haloperidol for 3 days and remained asymptomatic.

She also had a history of neurocysticercosis and convulsions since she was 14 years old. 

**Figure 3:**
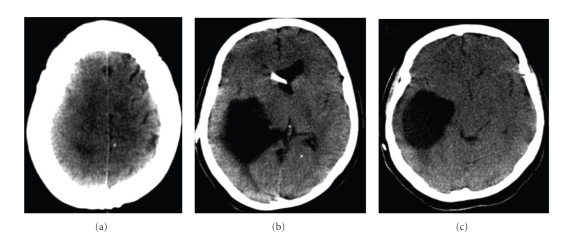
Noncontrast CT scans (2006). Ventriculo-peritoneal shunt with hypertensive obstructive hydrocephalus.

**Figure 4:**
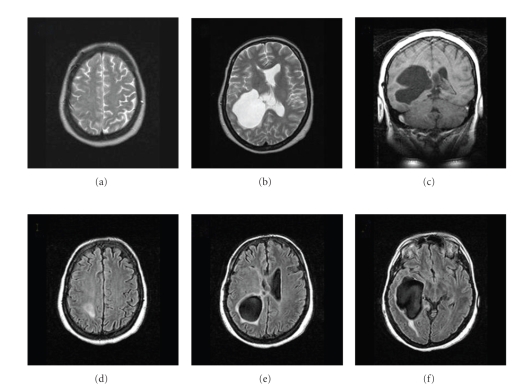
Magnetic resonance imaging (2006): (a) and (b) T2 weighted axial images; (c) T1 weighted coronal image; (d), (e), and (f) axial FLAIR (Fluid Attenuated Inversion Recovery) images. There are obstructive hydrocephalus and ventricular deformities, suggestive of intraventricular cystic lesions.

Computed tomography (Figure 3) showed an intraventricular cystic lesion and signs of intracranial hypertension. However, the patient did not show classical signs and symptoms of intracranial hypertension. Following examination with magnetic resonance imaging (Figure 4), endoscopic puncture of the cyst was indicated. 

**Figure 5:**
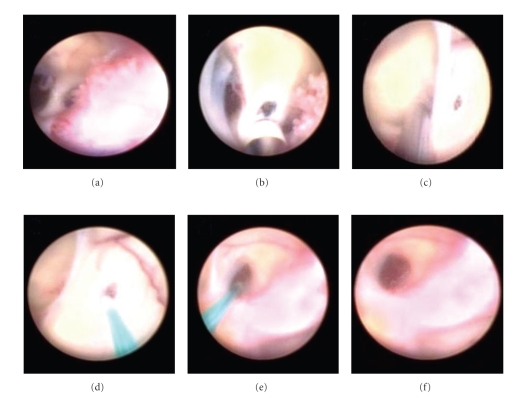
Screenshots of ventricular endoscopy (2006). Cystic walls were perforated, and the ventricular shunt was replaced.

**Figure 6:**
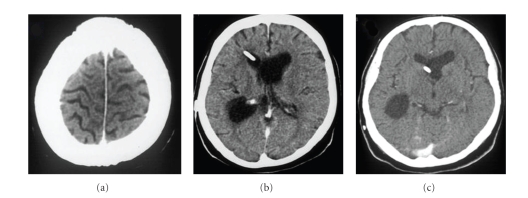
Noncontrast CT scans after endoscopic intraventricular cystic drainage and repositioning of the ventricular shunt (2006).

During the neurosurgery, an intraventricular fibrotic cyst was found and fenestrations were made to reestablish CSF flow. No evidence of active neurocysticercosis was found (Figure 5). The patient was discharged a week after the surgery with remission of the symptoms; she was prescribed valproate 1750 mg/d (Figure 6). When last contacted, in January, 2009, the patient was well and asymptomatic.

## 3. Discussion

This case describes a patient with manic and psychotic symptoms who had a history of neurocysticercosis and had presented with an episode of hypertensive hydrocephalus in 2003. Despite her history, she was initially treated for primary psychiatric disease.

Neurocysticercosis is the most common parasitic infection of the human CNS [1–3]. Its prevalence in rural areas of developing countries reaches 4% [4].

Hydrocephalus is one of the most frequent manifestations in neurocysticercosis, especially in the racemosus type, whose cysts occur preferentially in the cerebral ventricular system and basal cisterns [1, 3].

As no signs of active neurocysticercosis were found in this case, the cystic lesion constituted by fibrotic material may have been formed as inflammatory reaction caused by the degeneration of an intraventricular neurocysticercus. This lesion caused obstruction of the fluid flow and, consequently, hydrocephalus.

When evaluating a patient with psychiatric symptoms, it is essential to keep in mind the possibility of a general medical condition [5–7]. Although the association between hydrocephalus and manic symptoms is not frequent, it cannot be forgotten, even when classical hydrocephalus signs and symptoms are absent [8–10].
